# Evaluation of ceftazidime/avibactam for treatment of carbapenemase-producing carbapenem-resistant Enterobacterales with OXA-48 and/or NDM genes with or without combination therapy

**DOI:** 10.1093/jacamr/dlac104

**Published:** 2022-10-11

**Authors:** Hajar Alqahtani, Ahlam Alghamdi, Nouf Alobaidallah, Amal Alfayez, Rawan Almousa, Rawan Albagli, Nour Shamas, Fayssal Farahat, Ebrahim Mahmoud, Mohammad Bosaeed, Reem Abanamy

**Affiliations:** Department of Pharmaceutical Care, Ministry of National Guard Health Affairs, Riyadh, Saudi Arabia; Department of Pharmacy Practice, College of Pharmacy, Princess Nourah bint Abdulrahman University, Riyadh, Saudi Arabia; Department of Pharmaceutical Care, King Abdullah bin Abdulaziz University Hospital, Riyadh, Saudi Arabia; College of Pharmacy, Princess Nourah bint Abdulrahman University, Riyadh, Saudi Arabia; College of Pharmacy, Princess Nourah bint Abdulrahman University, Riyadh, Saudi Arabia; College of Pharmacy, Princess Nourah bint Abdulrahman University, Riyadh, Saudi Arabia; College of Pharmacy, Princess Nourah bint Abdulrahman University, Riyadh, Saudi Arabia; Department of Infection Prevention and Control, Ministry of National Guard, Health Affairs, Riyadh, Saudi Arabia; Department of Infection Prevention and Control, Ministry of National Guard, Health Affairs, Riyadh, Saudi Arabia; College of Public Health and Health Informatics, King Saud bin Abdulaziz University for Health Sciences, Riyadh, Saudi Arabia; Department of Medicine, Ministry of National Guard Health Affairs, Riyadh, Saudi Arabia; College of Medicine, King Saud bin Abdulaziz University for Health Sciences, Riyadh, Saudi Arabia; Department of Medicine, Ministry of National Guard Health Affairs, Riyadh, Saudi Arabia; College of Medicine, King Saud bin Abdulaziz University for Health Sciences, Riyadh, Saudi Arabia; Department of Clinical Trial Services, King Abdullah International Medical Research Center, Riyadh, Saudi Arabia; Department of Medicine, Ministry of National Guard Health Affairs, Riyadh, Saudi Arabia

## Abstract

**Background:**

Carbapenem-resistant Enterobacterales (CRE) is an urgent public health threat of significant global concern. Few observational studies have evaluated the clinical outcomes for treatment of CRE harbouring OXA-48 or NDM genes with ceftazidime/avibactam. Previous findings showed lower 30 day mortality with ceftazidime/avibactam ranges between 8.3% and 22%.

**Method:**

This single-centre retrospective cohort study included adult patients aged ≥18 years admitted to King Abdulaziz Medical City (KAMC) who had received ceftazidime/avibactam for at least 72 h for infections caused by CRE with genes encoding for carbapenemase production (CP-CRE).

**Results:**

A total of 211 patients, mostly male (57%), having CP-CRE infections treated with ceftazidime/avibactam were included, with an average age of 62 years. More than 50% of patients were critically ill, for which 46% received invasive ventilation and 36% were on inotropes. The most frequent infectious disease was hospital/ventilator-acquired pneumonia with *Klebsiella pneumoniae* being the most frequent causative pathogen. The majority of isolates harboured OXA-48 (81%), followed by NDM ± OXA-48 (19%). The overall clinical cure and 30 day mortality was 78% and 21% respectively (stratified per gene: 79% and 21.6% for OXA-48 and 75% and 17.5% for NDM ± OXA-48).

**Conclusions:**

This was the largest study that evaluated clinical outcomes associate with CP-CRE harbouring OXA-48 gene infections treated with ceftazidime/avibactam. Clinical cure and 30 day mortality were consistent with those of previous studies. Findings suggested that combination therapy with ceftazidime/avibactam had no direct impact on clinical outcomes for CP-CRE with OXA-48.

## Introduction

Carbapenem-resistant Enterobacterales (CRE) is an urgent public health threat of significant global concern that accounted for more than 1100 deaths per the 2019 CDC report for antibiotic resistance.^1^ CRE is associated with higher mortality rate, with an approximately 2-fold increase in mortality compared with infections with carbapenem-susceptible Enterobacterales.^2^ Carbapenemases produced by CRE have been classified into three different groups according to Ambler classification: class A [e.g. *Klebsiella pneumoniae* carbapenemase (KPC)], class B or MBL (e.g. VIM) and class D [e.g. oxacillinase (OXA-48)].^2,3^ Local data that describe the molecular epidemiology of CRE in Saudi Arabia found that OXA-48 accounted for 71.2% (*n* = 292) of 410 carbapenemase-producing CRE isolates, followed by NDM (*n* = 85; 20.7%) and NDM + OXA-48 (*n* = 33; 8%).^4^

The new β-lactam/β-lactamase inhibitors have gained significant interest for their favourable outcomes in terms of clinical efficacy and safety profile.^5^ Ceftazidime/avibactam is a combination of a third-generation cephalosporin, ceftazidime, and the novel, non-β-lactam β-lactamase inhibitor avibactam, which possesses the ability to treat carbapenemase-producing CRE (CP-CRE).^5^ Ceftazidime/avibactam was approved by the FDA in 2015 for complicated urinary tract infections (cUTIs), complicated intra-abdominal infections (cIAIs) and, in 2018, for the treatment of hospital-acquired pneumonia (HAP) and ventilator-associated pneumonia (VAP).^6^

Few observational studies have evaluated the clinical outcomes for treatment of CP-CRE harbouring OXA-48 with ceftazidime/avibactam.^7,8^ Findings of these studies showed lower 30 day mortality with ceftazidime/avibactam ranging between 8.3% and 22%.^7,8^ Notably, the small sample size of both studies would affect its generalizability and increase the value in other studies with larger sample sizes.

Considering the high prevalence of Gram-negative resistant pathogens, particularly CP-CRE, at our institution,^9^ the aim of this study was to evaluate the clinical efficacy of ceftazidime/avibactam for treatment of CP-CRE harbouring OXA-48 and/or NDM.

## Methods

### Study design and setting

This was a retrospective, observational, single-centre study conducted at King Abdulaziz Medical City (KAMC) from January 2018 to November 2020. KAMC is a Joint Commission International (JCI)-accredited 1500-bed tertiary care academic medical centre in Riyadh, Saudi Arabia.

### Study population

The inclusion criteria of patients were if they were adults (age ≥18 years) and had received ceftazidime/avibactam alone or in combination for ≥72 h as treatment of a confirmed infection secondary to CP-CRE with a detected gene encoding for carbapenemase production. At least one isolate was collected and isolated from each case for microbiological analysis. All patients were stratified into two groups: those who had received ceftazidime/avibactam alone (monotherapy group) and those who had combination therapy added to ceftazidime/avibactam for treatment of CP-CRE as per definition (combination therapy group). Patients with polymicrobial infections and those who did not receive aztreonam in combination with ceftazidime/avibactam for CP-CRE encoded with NDM were excluded. The study was approved by the institutional review board at King Abdullah International Medical Research Center (KAIMRC). Clinical data were collected from electronic medical records and de-identified for further review. Relevant information, including demographic data, baseline comorbidities, Charlson comorbidity index, infection and treatment-related variables, was recorded.

### Microbiological tests

Microbiological tests for bacterial identification and susceptibility testing were performed in the laboratory department in accordance with CLSI protocols. Susceptibility tests were conducted by an automated susceptibility testing system via the VITEK 2 system (bioMérieux, Marcy-l’Étoile, France). Per local microbiology lab protocol, isolates were subjected for further work-up when susceptibility tests showed resistance to ertapenem, meropenem or imipenem/cilastatin, defined as when MICs are at the resistance cut-off of the susceptibility breakpoints determined by CLSI protocol (≥1 mg/L for meropenem and imipenem/cilastatin and ≥0.5 mg/L for ertapenem). Such isolates would undergo PCR-based testing to identify the presence of any gene coding for carbapenemase production and thus classified as CP-CRE. Ceftazidime/avibactam MICs were determined by Etest (bioMérieux). Broth microdilution was used to determine colistin MICs. The results were interpreted in accordance with the CLSI clinical breakpoints (≤2 mg/L interpreted as susceptible or intermediately susceptible, after recent updates of CLSI in 2020). Carbapenemase gene content was detected using the GeneXpert Carba-R PCR assays (Cepheid, Sunnyvale, CA, USA) targeting *bla*_KPC_, *bla*_OXA-48-like_, *bla*_VIM_, *bla*_NDM_ and *bla*_IMP_.

### Antimicrobial therapy

Ceftazidime/avibactam is a restricted medication at KAMC for critical care, haematology/oncology and infectious disease (ID), and it can be dispensed for 72 h without an ID approval. Therapy with ceftazidime/avibactam was usually started by the primary treating physician, which was often followed by an ID consultation. Dosage set was 2.5 g every 8 h to be infused over 2 h. Other antibiotics that were frequently used as a combination therapy for CP-CRE were colistin, amikacin, gentamicin, tigecycline and aztreonam.

### Definitions

The infection source was determined based on the CDC/National Healthcare Safety Network criteria. Treatment regimens were classified as monotherapy (treatment with ceftazidime/avibactam alone) or combination therapy, defined as the addition of other IV antimicrobials with *in vitro* activity against the clinical isolate or the addition of aztreonam to ceftazidime/avibactam for isolates with NDM genes detected. Concomitant treatment with metronidazole (when required for anaerobic coverage) was not considered as combination therapy. Acute kidney injury (AKI) was defined as an increase in serum creatinine by 0.3 mg/dL over 48 h or a reduction in urine output to 0.5 mL/kg/h over 6 h.

### Outcomes

Primary outcomes were determined to be clinical cure at completion of therapy and 30 day mortality rate. For the secondary outcomes, 60 day mortality and relapse rate within 30 days of completion of ceftazidime/avibactam therapy for primary infection were determined. Safety outcomes include development of adverse drug reactions related to ceftazidime/avibactam. Clinical cure was defined as clinical improvement with no signs or symptoms of systemic infection (absence of fever, leucocytosis and elevated inflammatory markers) at the completion of therapy. Relapse was defined as reinfection with the same organism within 30 days after completion of ceftazidime/avibactam therapy.

### Statistical analysis

Categorical variables were expressed as percentages and compared between groups using the chi-squared test or Fisher’s exact test based on the data distribution. Continuous variables were presented as the means and standard deviation. Student’s *t*-test or Wilcoxon/Kruskal–Wallis tests were used to compare the groups for continuous variables based on the data distribution. Further, we used multivariate logistic regression analysis to model the 30 day and 60 day mortality as outcomes. We included patients: with BMI ≥30 kg/m^2^, with diabetes mellitus (DM), hypertension (HTN), pneumonia, bacteraemia, UTIs, IAIs, patients infected with CRE encoded by OXA-48 versus NDM, who received vasopressors or required mechanical ventilation (MV), who received ceftazidime/avibactam as monotherapy or in combination, who received the appropriate dosing of ceftazidime/avibactam versus those who did not, with CL_CR_ ≥ 50 mL/min versus <50 mL/min, who received ceftazidime/avibactam for ≥7 days versus ≥10 days versus ≥14 days, with a history of CRE infection in the last year before current incidence; patient’s age and sex were predictors with backward elimination, with *P* = 0.5 for entry and 0.05 to stay in the model.

## Results

**Figure 1.**
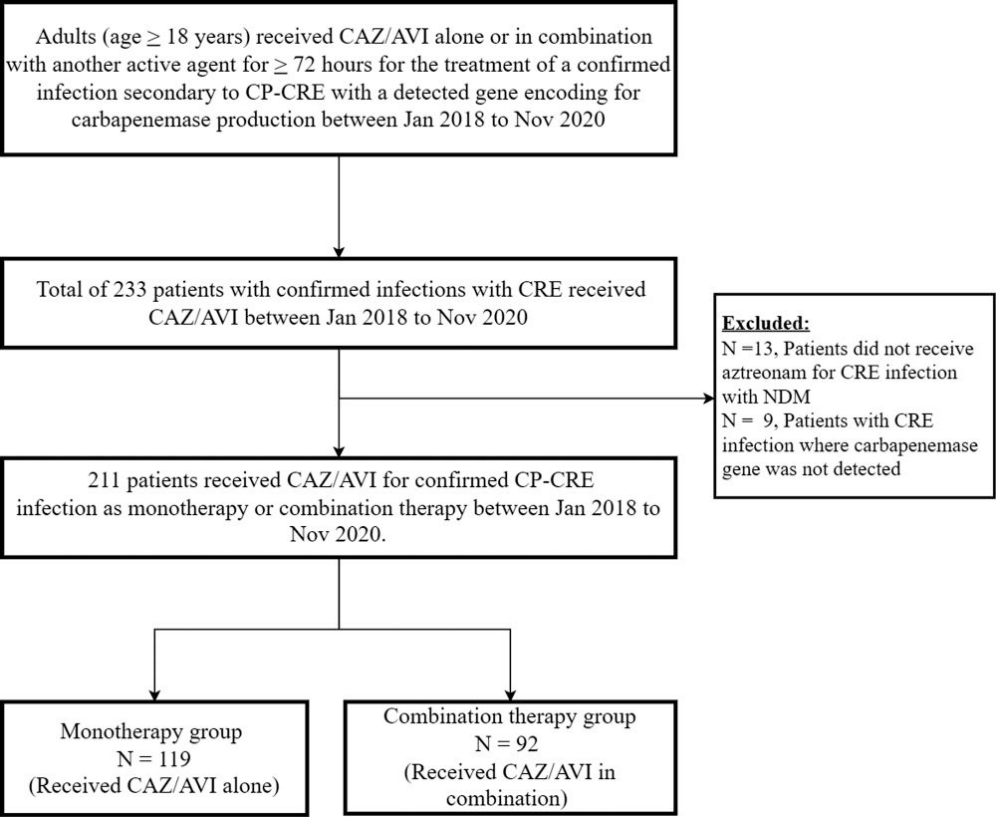
Patient allocation. CAZ/AVI, ceftazidime/avibactam.

A total of 233 confirmed infections with CRE were identified between January 2018 and November 2020. Twenty-two patients were excluded for not fully meeting the inclusion criteria. A total of 211 patients received ceftazidime/avibactam for CP-CRE infection during the study period (Figure 1).

For those who received ceftazidime/avibactam as monotherapy (*n* = 119), the average age was 66 years and the majority of these were male (55.6%). Combination therapy was prescribed for 92 patients (61% male) with an average age of 56 years. HTN and DM followed by heart disease were the most frequently seen comorbidities in monotherapy and combination therapy groups [(70% versus 48%; *P* = 0.015); (69% versus 49%; *P* = 0.031); (54% versus 48%; *P* = 0.259)], respectively. There were 61 (51.3%) patients with a Charlson index score of 5 or higher in the monotherapy group and 36 (39%) in the combination group (*P* = 0.08). Infection biochemical markers, including leucocytosis, elevated procalcitonin, high erythrocyte sedimentation rate and C-reactive protein, were elevated in both groups with no statistically significant differences (Table 1).

**Table 1.** Baseline characteristics

	Monotherapy *N* = 119	Combination therapy *N* = 92	Total *N* = 211	*P* value
Demographic data				
* *Age, years, mean ± SD	66 ± 18	56 ± 19	62 ± 19	0.005
* *Female, *n* (%)	54 (45.4)	36 (39)	90 (43)	0.962
* *Weight, kg, mean ± SD	73 ± 18	74 ± 17	73 ± 18	0.376
* *BMI, kg/m^2^, mean ± SD	28 ± 7	28 ± 7	28 ± 7	0.845
Medical history—baseline comorbidities, *n* (%)				
* *Heart disease	64 (54)	44 (48)	108 (51)	0.259
* *DM	82 (69)	45 (49)	127 (60)	0.031
* *Hypertension	83 (70)	44 (48)	127 (60)	0.015
* *Chronic kidney disease	32 (27)	21 (23)	53 (25)	0.757
* *Haemodialysis	20 (17)	11 (12)	31 (11.4)	0.516
* *Liver disease	15 (13)	9 (10)	24 (15)	0.554
* *Respiratory disease	24 (20)	16 (17)	40 (19)	0.73
* *Active malignancy	12 (10)	16 (17)	28 (13.3)	0.417
* *Charlson index score ≥5	61 (51.3)	36 (39)	97 (46)	0.08
Baseline laboratory parameters on the day where positive culture for CRE was obtained				
* *WBCs (10^9^/L)	12 ± 6	13 ± 10	12 ± 8	0.366
* *Neutrophils (cells/mm^3^)	7 ± 6	9 ± 8	8 ± 5	0.07
* *Procalcitonin (ng/mL)	6 ± 14	11 ± 34	9 ± 29	0.303
* *Lactic acid (mmol/L)	2.4 ± 2	2 ± 1	2 ± 2	0.215
* *Erythrocyte sedimentation rate (mm/hour)	53 ± 31	58 ± 36	61 ± 35	0.264
* *C-reactive protein (mg/L)	139 ± 181	133 ± 85	128 ± 170	0.972
* *AST (units/L)	35 ± 34	57 ± 54	64 ± 111	0.307
* *ALT (units/L)	53 ± 92	50 ± 60	50 ± 78	0.524
Critically ill patients, *N* = 132, *n* (%)				
* *ICU at index culture	51 (43)	56 (61)	107 (51)	0.025
* *MV	47 (39)	50 (55)	97 (46)	0.021
* *Vasopressors	39 (32.8)	42 (46)	81 (38)	0.051
* *Continuous renal replacement therapy	4 (3.4)	6 (6.5)	10 (4.7)	0.773
Microbiological findings and ID diagnosis				
* *Specimens collected, *n* (%)				
* *Respiratory	37 (31)	29 (32)	66(31.3)	0.29
* *Urine	37 (31)	21 (23)	58 (27.5)	
* *Blood	26 (22)	28 (30)	54(25.6)	
* *Deep wound swabs	7 (6)	15 (16.3)	22(10.4)	
* *Fluid culture	11 (9.3)	4 (4.3)	15(7)	
* *Tissue	8 (7)	6 (6.5)	13 (6)	
* *Isolated organisms, *n* (%)				
* K. pneumoniae*	110 (92)	79 (86)	189 (90)	0.216
* E. coli*	4 (3.4)	5 (5.3)	9 (4.3)	
* Enterobacter* spp.	6 (5)	3 (3.3)	9 (4.3)	
* Klebsiella oxytoca*	0	1 (1)	1 (0.5)	
* Proteus mirabilis*	1 (0.8)	0	1 (0.5)	
* Citrobacter* spp.	0	1 (1)	1 (0.5)	
* Serratia marcescens*	0	3 (3.3)	3 (1.4)	
* *Gene encoding carbapenemase enzyme, *n* (%)				
* *OXA-48	119 (100)	52 (56.5)	171 (81)	0.001
* *NDM	0	29 (31.5)	29 (13.7)	0.001
* *Both (OXA-48 + NDM)	0	11 (12)	11 (5.2)	
* *ID diagnosis, *n* (%)				
* *HAP/VAP	37 (31)	29 (32)	66(31.3)	0.55
* *UTI	36 (30.3)	20 (21.7)	56 (26.5)	
* *Bacteraemia	26 (22)	28 (30.4)	54(25.6)	
* *CRBSI	6 (23)	5 (17.9)	11 (20)	
* *IAI	11 (9)	4 (4.3)	15 (7)	
* *SSTI, osteomyelitis, SSI	14 (11.8)	22 (24)	36 (17)	
* *PJI, infected hardware	3 (2.5)	3 (3.3)	6 (2.8)	
* *CAZ/AVI therapy information				
* *Initial dose, *n* (%)				
* *2500 mg	69	66	135 (64)	0.67
* *1250 mg	21	8	29 (13.7)	
* *940 mg	29	18	47 (22.3)	
* *Maintenance dose, *n* (%)				
* *2500 mg	63	72	135 (64)	0.87
* *1250 mg	19	15	34 (16.1)	
* *940 mg	37	15	52 (25)	
* *Frequency, *n* (%)				
* *q8h	82	76	158 (75)	0.69
* *q12h	20	10	30 (14.2)	
* *q24h	13	4	17 (8)	
* *q48h	4	2	6 (3)	
* *CL_CR_ at initiation, mL/min, mean ± SD	64 ± 47	84 ± 54	74 ± 51	0.019
* *Appropriate dose, *n* (%)	93 (78)	71 (77)	164 (77.7)	0.55
* *Time from identifying CRE until CAZ/AVI initiation, days, median	1	1	1	0.334
* *Adequate source control, *n* (%)	49 (42)	41 (45)	90 (43)	0.349
* *Duration of therapy, days, mean (SD)	14 ± 10	17 ± 15	16 ± 13	0.08
Combination therapy information				
* *Combination agent, *n* (%)				
* *Colistin	0	25 (27)	25 (11.8)	
* *Tigecycline	0	16 (17.4)	16 (8)	
* *Amikacin	0	10 (11)	10 (5)	
* *Aztreonam	0	41 (45)	41 (19)	
* *Gentamicin	0	18 (20)	18 (9)	

CRBSI, central line-related bloodstream infection; SSTI, skin/soft tissue infection; SSI, surgical site infection; PJI, prosthetic joint infection; CAZ/AVI, ceftazidime/avibactam.

Compared with the monotherapy group, more patients in the combination group were in ICUs when cultures were collected (61% versus 43%; *P* = 0.025). The number of patients receiving invasive MV and vasopressors was significantly higher in the combination group compared with the monotherapy group (55% versus 39%; *P* = 0.021 and 46% versus 32.8%; *P* = 0.051, respectively).

**Figure 2.**
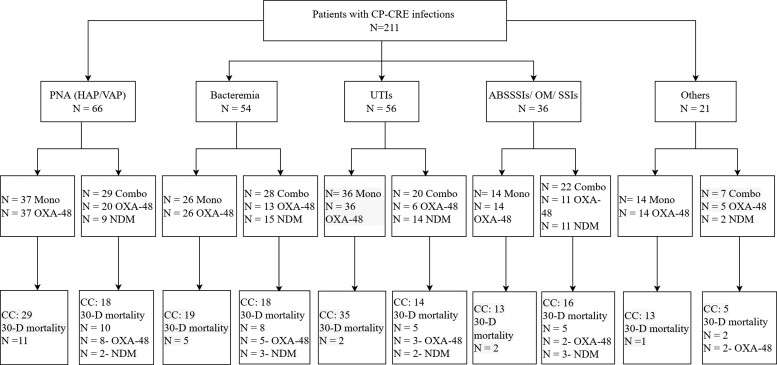
Final disposition of patients with CP-CRE stratified by ID diagnosis. PNA, pneumonia; ABSSSI, acute bacterial skin and skin structure infection; OM, osteomyelitis; SSI, surgical site infection; Others includes intra-abdominal infection, infected hardware, prosthetic joint infection; CC, clinical cure; 30-D, 30 day.

The most frequently isolated pathogens from 211 patients were *K. pneumoniae* (189/211; 90%), *Escherichia coli* (9/211, 4.3%) and *Enterobacter* spp. (9/211; 4.3%). The majority of collected specimens were from the respiratory tract (sputum, tracheal aspirate, bronchoalveolar lavage) (31.3%), followed by urine (27.5%) and blood (25.6%). More than 80% of isolates (171/211; 81%) carried OXA-48 genes, followed by NDM ± OXA-48 (40/211; 19%). Ceftazidime/avibactam susceptibility was done for 76 isolates (44 and 32 in the monotherapy group and combination groups, respectively); these were all susceptible and carried the OXA-48 gene. Pneumonia (HAP and VAP) was the most frequently encountered ID (31.3%) followed by UTIs (26.5%) and bacteraemia (25.6%) (Figure 2). The appropriateness of ceftazidime/avibactam dosing was observed in 77.7% of the total cohort; however, 6% received higher doses since they were determined to have resolved AKI secondary to sepsis, and 17% received lower doses than recommended. The median time between identifying CP-CRE and ceftazidime/avibactam initiation was 24 h in both groups. The mean duration of ceftazidime/avibactam therapy was prolonged in combination therapy (17 versus 14 days; *P* = 0.08). Combination therapy was prescribed for 92 patients, 45% of whom received aztreonam; 27%, colistin; 20%, gentamicin; and the remaining received tigecycline (17.4%) and amikacin (11%) (Table 2).

**Table 2.** Outcomes of interest

Clinical outcomes				
	Monotherapy, *N* = 119 (OXA-48 = 119)	Combination therapy, *N* = 92 (OXA-48 = 52; NDM = 40)	Total, *N* = 211	*P* value
Clinical cure, *n* (%)	103 (87)	62 (67.4)	165 (78.2)	0.001
* *Isolates with OXA-48 gene	103 (87)	32 (61.5)	135 (79)	0.0001
* *Isolates with NDM ± OXA-48 genes	0	30 (75)		
30 day mortality, *n* (%)	19 (16)	25 (27)	44 (21)	0.05
* *Isolates with OXA-48 gene	19 (16)	18 (34.6)	37 (21.6)	0.006
* *Isolates with NDM ± OXA-48 genes	0	7 (17.5)		
60 day mortality, *n* (%)	24 (20.2)	33 (36)	57 (27)	0.011
* *Isolates with OXA-48 gene	24 (20.2)	23 (44)	47 (27.5)	0.001
* *Isolates with NDM ± OXA-48 gene	0	10 (25)		
Relapse within 30 days of completion of CAZ/AVI course, *n* (%)	9 (7.6)	12 (13)	21 (10)	0.19
* *Isolates with OXA-48 gene	9 (7.6)	6 (11.5)	15 (8.8)	0.42
* *Isolates with NDM ± OXA-48 genes	0	6 (15)		
Adverse drug reactions, *n* (%)				
* *AKI related to antimicrobial agent	8 (6.7)	8 (8.7)	16 (8)	0.652
* C. difficile* infection	5 (4.2)	4 (4.3)	9 (4)	0.982
* *Rash resulting in discontinuing CAZ/AVI	1 (0.8)	0	1(0.5)	
* *Liver function tests elevated (AST/ALT)	4 (3.4)	4 (4.3)	8 (3.8)	
Development of invasive fungal infection, *n* (%)	28 (23.5)	19 (21)	47 (22.8)	0.955

### Outcomes of interest

Clinical cure was significantly higher in the monotherapy group compared with the combination therapy group (87% versus 67.4%; *P* = 0.001). The mortality rate at 30 days post infection was 16% and 27% (*P* = 0.05) in monotherapy and combination therapy groups, respectively; for secondary outcomes, 60 day mortality rates were 20.2% and 36%, respectively, which was a significant difference (*P* = 0.011). Relapse rates of infection with CR-CRE at 30 days post ceftazidime/avibactam therapy completion were 7.6% and 13% in monotherapy and combination therapy, respectively (Table 2).

In the monotherapy group, 6.7% of patients developed AKI compared with 8.7% in the combination group. Almost 4% of patients in each group developed *Clostridioides difficile* diarrhoea and 20% developed candidaemia.

**Figure 3.**
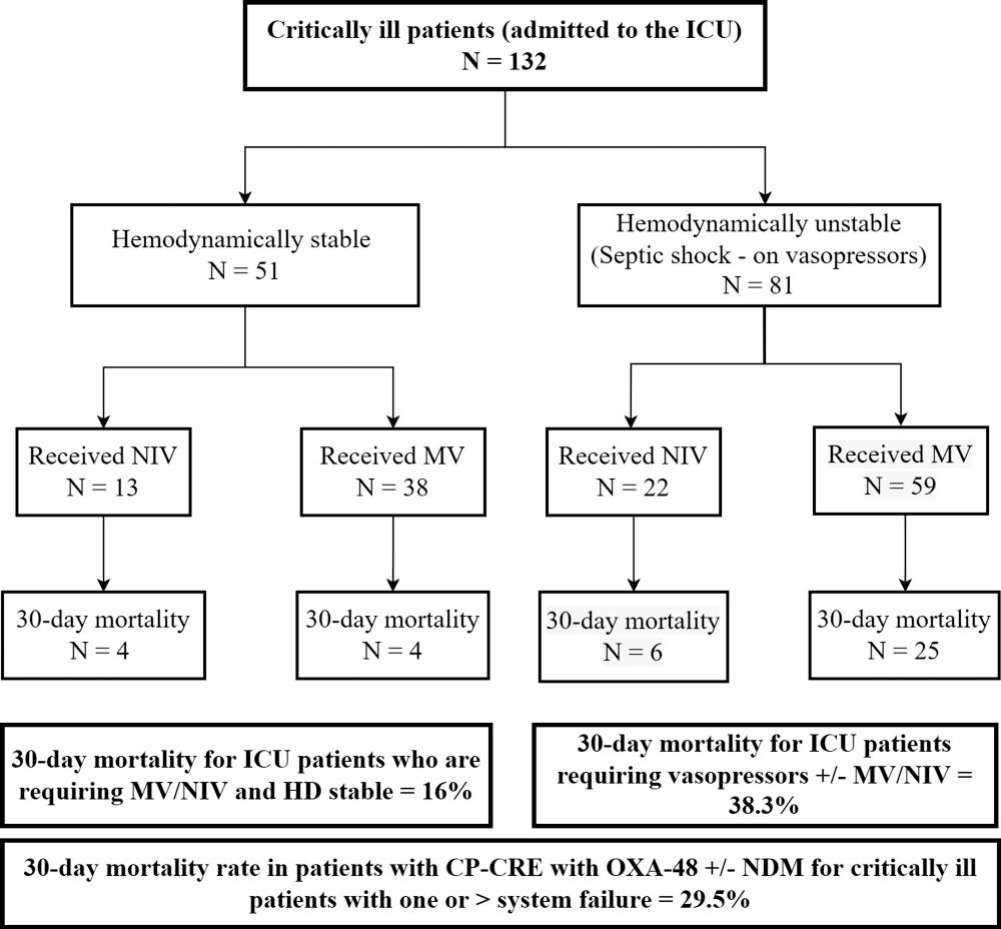
Illustration of final disposition of critically ill patients with CP-CRE. NIV, non-invasive ventilation; HD, haemodynamically.

Multivariate logistic regression analysis showed statistically significant associations between different variables and 30 day and 60 day mortality rates. Septic shock at the time of infection was associated with an increased risk of 30 day and 60 day mortality by 7-fold and 5-fold, respectively (Table 3, Figure 3).

**Table 3.** Multivariate logistic regression analysis for 30 day and 60 day mortality

Variables	30 day mortality		60 day mortality	
	OR (95% CI)	*P*	OR (95% CI)	*P* value
Charlson score	1.38 (1.15–1.65)	0.001		
BMI ≥ 30 kg/m^2^	2.2 (0.95–4.8)	0.066	3.25 (1.01–10.4)	0.047
Pneumonia	3.2 (1.35–7.48)	0.008		
Active malignancy	3.43 (1.16–10.04)	0.026	4.9 (0.79–30.3)	0.087
Septic shock	7.2 (3.01–17.3)	0.001	4.9 (1.22–19.8)	0.025
Bacteraemia			6 (1.38–25.87)	0.017
Duration of therapy ≥7 days	0.3 (0.10–0.85)	0.025	0.12 (0.01–1.23)	0.074
Appropriate dose	0.32 (0.14–0.77)	0.011		
History of infection with CRE in last year	0.14 (0.05–0.43)	0.001		

Patients with BMI ≥30 kg/m^2^ and active malignancy were at higher risk of mortality with OR = 2.2 (95% CI 0.95–4.8; *P* = 0.07) and 3.43 (95% CI 1.16–10.04; *P* = 0.03).

The appropriate dosing and duration of ≥7 days of ceftazidime/avibactam was associated with a 30% decreased risk of 30 day mortality. Interestingly, patients with a history of infection with CRE within the last year before the current infection were associated with a 20% lower risk of 30 day mortality (Table 3).

Combination therapy may be associated with more severe infection, a potential confounder of poor clinical outcomes. Therefore, a propensity score was calculated, and multiple regression analysis showed that adjusted OR for combination therapy in association with 30 day mortality was 1.5 (95% CI 0.61–3.64); *P* = 0.38, and in association with 60 day mortality was 1.24 (95% CI 0.35– 4.42); *P* = 0.75.

## Discussion

To the best of our knowledge, this is the largest study that has evaluated ceftazidime/avibactam efficacy and safety in infections secondary to CP-CRE with OXA-48 genes. Our study included 171 patients with infections caused by CP-CRE with OXA-48 genes and 40 patients with NDM ± OXA-48. Clinical cure and 30 day mortality rate among patients with infections caused by CP-CRE with OXA-48 ± NDM genes treated with ceftazidime/avibactam were 78% and 21%, respectively. In particular, 79% of patients achieved clinical cure and 21.6% died within 30 days of the onset of CP-CRE infection with OXA-48. Our results are consistent with those reported by *Sousa et al.*,^7^ where 30 day mortality was 22.8% among a cohort of 57 patients with CP-CRE OXA-48 and a clinical cure rate of 77%. Local data from Saudi Arabia have evaluated ceftazidime/avibactam for treatment of infection with CP-CRE OXA-48 and found a 30 day mortality rate of 20% (2/10) compared with 39.3% (11/28) for patients treated with other agents.^10^ De la Calle *et al.*^8^ showed a lower mortality rate (8.3%; 2/24) for patients having CP-CRE with OXA-48 infection treated with ceftazidime/avibactam. Certainly, small sample size would potentially over- or under-estimate the impact of ceftazidime/avibactam, as in all previously mentioned studies.^7,8,10^ Evidently, cumulative data consistently proved the association of lower mortality rate and higher clinical cure with treatment of CP-CRE encoded with the OXA-48 gene with ceftazidime/avibactam compared with best available options.^7,8,10^ Of note, in this study, the 30 day mortality among 40 patients who received a combination of ceftazidime/avibactam and aztreonam for infections caused by CP-CRE with NDM ± OXA-48 genes was 17.5%, with a clinical cure rate of 75%, which is consistent with those reported by *Falcone et al*.^11^ Although our patient cohort is considered small, it clearly highlights the efficacy of combining ceftazidime/avibactam with aztreonam for CP-CRE with NDM ± OXA-48 genes by achieving results consistent with *Falcone et al*.^11^ Multivariate logistic regression analysis revealed factors independently associated with higher 30 day mortality included high Charlson score, patients with HAP/VAP, active malignancy and septic shock at presentation. It is noteworthy that our study was conducted during the first wave of COVID-19 in Saudi Arabia, which could have contributed to the increased risk of death among patients with VAP. Moreover, the 30 day mortality rate is well known to be higher in patients with active malignancies and infections with CRE compared with those without active malignancies.^12,13^

A 7 day duration of ceftazidime/avibactam therapy and appropriate dosing have been associated with a 30% reduction in 30 day mortality. Interestingly, patients with a history of previous infection with CRE are at 20% lower risk of death within 30 days of infection. Our study is the first to address such an association, which has not been rationalized before. It is out of the scope of this study to elaborate on this association; however, this could open doors for future discussion on the impact of subsequent infections of CRE and immunity memory formation, therefore exhibiting a better response manifested in improved survival rate. Combination therapy with ceftazidime/avibactam for treatment of CP-CRE with OXA-48 seems to lack positive impact on survival, clinical cure and relapse. This finding is consistent with other studies on CP-CRE with OXA-48 and CP-CRE with KPC.^7,10,14^ Relapse within 30 days post completion of ceftazidime/avibactam was 10% among our cohort, agreeing with results from Hakem *et al*.^15^

The favourable safety profile of ceftazidime/avibactam**-**based therapy is one of the properties that has helped ceftazidime/avibactam to replace other agents and gain more attention in clinical practice. Our results demonstrated a lower rate of AKI (8%) during ceftazidime/avibactam therapy compared with local data.^15,16^ Moreover, the majority of the 25% of our patients who received nephrotoxic antimicrobial agents for a mean duration of 13 days were critically ill. AKI in such patients is multifactorial and cannot be correlated with a single aetiology.

### Conclusions

This study was the largest study to evaluate clinical outcomes associated with CP-CRE harbouring OXA-48 infections treated with ceftazidime/avibactam. Clinical cure and 30 day mortality were consistent with those of previous studies. The findings suggested that combination therapy with ceftazidime/avibactam had no direct impact on clinical outcomes. The patients who presented with septic shock appeared to be at the highest risk of mortality.

## Data Availability

Datasets are available upon request from the first author (H.A.; Hajar.alqahtani99@gmail.com) on reasonable request, as long as this meets local ethics and research government criteria.
